# Supplementary material for: A Mendelian randomization study of the effects of blood lipids on breast cancer risk
Source: Nat Commun. 2018 Sep 27;9:3957. doi: 10.1038/s41467-018-06467-9 (PMC6160471; doi:10.1038/s41467-018-06467-9)
Supplement: Supplementary file 1 — Supplementary Information [file 41467_2018_6467_MOESM1_ESM.pdf]

Supplementary Information

A Mendelian randomization study of the effects of blood lipids on breast cancer risk

Nowak et al.

Supplementary Table 1

Genetic variants used in instrumental variable analysis for blood lipids.

Genetic variants used in instrumental variable analysis for blood lipid levels

rsid	chr	position_b37	effect_ allele	other_ allele	b_ldl	se_ldl	p_ldl	b_hdl	se_hdl	p_hdl	b_tg	se_tg	p_tg	b_bca	se_bca	p_bca	b_erpos	se_erpos	p_erpos	b_erneg	se_erneg	p_erneg
rs10019888	4	26,062,990	G	A	1.82E-02	5.00E-03	3.23E-04	-2.70E-02	4.60E-03	4.90E-08	2.28E-02	4.50E-03	2.28E-06	4.90E-03	8.60E-03	5.69E-01	1.59E-02	1.03E-02	1.21E-01	-8.00E-03	1.57E-02	6.10E-01
rs10029254	4	88,160,140	T	C	5.80E-03	4.40E-03	2.05E-01	-8.50E-03	4.10E-03	4.87E-02	2.66E-02	3.90E-03	7.55E-09	4.60E-03	7.40E-03	5.36E-01	6.00E-03	8.80E-03	4.98E-01	2.40E-03	1.35E-02	8.61E-01
rs1010167	1	110,198,727	G	C	2.50E-02	3.90E-03	6.22E-11	-4.40E-03	3.70E-03	3.96E-01	1.60E-03	3.50E-03	8.08E-01	1.22E-02	6.40E-03	5.79E-02	1.28E-02	7.60E-03	9.31E-02	2.20E-02	1.16E-02	5.85E-02
rs10102164	8	55,421,614	A	G	3.16E-02	4.50E-03	3.74E-11	-5.00E-04	4.20E-03	7.97E-01	1.13E-02	4.10E-03	6.87E-03	4.00E-03	7.80E-03	6.12E-01	-1.00E-04	9.30E-03	9.94E-01	-3.40E-03	1.43E-02	8.11E-01
rs10282707	7	17,911,038	T	C	-8.40E-03	3.80E-03	4.23E-02	-2.50E-02	3.50E-03	1.03E-11	9.20E-03	3.40E-03	6.52E-03	-2.80E-03	6.60E-03	6.66E-01	-8.80E-03	7.80E-03	2.60E-01	3.50E-03	1.19E-02	7.66E-01
rs103294	19	54,797,848	T	C	7.30E-03	4.70E-03	1.23E-01	5.23E-02	4.40E-03	4.00E-30	-2.10E-03	4.20E-03	7.52E-01	-6.30E-03	7.90E-03	4.23E-01	-9.00E-04	9.40E-03	9.27E-01	-1.50E-03	1.45E-02	9.18E-01
rs1035744	15	72,566,615	T	C	6.90E-03	4.10E-03	1.58E-01	-5.50E-03	3.80E-03	1.55E-01	2.07E-02	3.80E-03	1.45E-07	-4.00E-03	7.00E-03	5.61E-01	-1.28E-02	8.30E-03	1.22E-01	7.00E-03	1.27E-02	5.81E-01
rs10401969	19	19,407,718	C	T	-1.18E-01	7.20E-03	2.65E-54	1.28E-02	6.80E-03	1.02E-01	-1.21E-01	6.50E-03	9.70E-70	1.57E-02	1.15E-02	1.72E-01	2.27E-02	1.38E-02	9.84E-02	2.01E-02	2.11E-02	3.40E-01
rs1047891	2	211,540,507	A	C	7.90E-03	4.20E-03	1.42E-01	-2.69E-02	3.90E-03	8.73E-10	1.00E-06	3.80E-03	8.60E-01	1.06E-02	7.00E-03	1.28E-01	7.00E-04	8.20E-03	9.33E-01	3.61E-02	1.26E-02	4.23E-03
rs10493326	1	62,953,373	A	G	2.13E-02	4.30E-03	1.91E-06	-1.30E-03	4.00E-03	6.73E-01	3.11E-02	3.90E-03	2.00E-15	-5.00E-04	7.20E-03	9.43E-01	-6.80E-03	8.60E-03	4.30E-01	1.50E-02	1.32E-02	2.55E-01
rs10513688	3	170,727,218	A	G	2.22E-02	6.10E-03	2.18E-03	-4.90E-03	5.70E-03	6.08E-01	3.06E-02	5.60E-03	1.54E-07	1.04E-02	1.04E-02	3.17E-01	3.00E-03	1.24E-02	8.09E-01	1.45E-02	1.89E-02	4.42E-01
rs10773105	12	125,283,766	T	C	5.80E-03	3.70E-03	1.22E-01	-3.63E-02	3.50E-03	3.20E-24	3.70E-03	3.40E-03	5.09E-01	2.50E-03	6.20E-03	6.92E-01	-5.50E-03	7.40E-03	4.56E-01	1.08E-02	1.13E-02	3.37E-01
rs10790162	11	116,639,104	G	A	-7.60E-02	7.20E-03	1.09E-23	9.51E-02	6.70E-03	9.91E-40	-2.31E-01	6.50E-03	1.10E-249	4.70E-03	1.20E-02	6.98E-01	6.00E-03	1.43E-02	6.74E-01	-1.38E-02	2.17E-02	5.26E-01
rs10832962	11	18,656,271	T	C	3.20E-02	4.00E-03	6.62E-14	4.30E-03	3.80E-03	3.33E-01	1.09E-02	3.70E-03	5.18E-03	3.18E-02	7.40E-03	1.78E-05	3.81E-02	8.90E-03	1.70E-05	4.45E-02	1.35E-02	9.85E-04
rs10861661	12	107,174,646	C	A	4.00E-04	4.50E-03	9.29E-01	-2.20E-02	4.20E-03	5.05E-07	2.27E-02	4.10E-03	2.60E-07	-5.90E-03	7.40E-03	4.25E-01	-1.07E-02	8.90E-03	2.30E-01	1.70E-03	1.36E-02	8.98E-01
rs10903129	1	25,768,937	G	A	3.28E-02	3.70E-03	3.03E-17	9.00E-04	3.40E-03	8.59E-01	8.00E-03	3.30E-03	5.88E-03	-1.37E-02	6.20E-03	2.74E-02	-6.70E-03	7.50E-03	3.73E-01	-1.91E-02	1.13E-02	9.11E-02
rs11045163	12	20,463,526	G	A	-5.50E-03	3.70E-03	1.63E-01	2.17E-02	3.50E-03	3.20E-09	-9.70E-03	3.40E-03	2.85E-03	3.80E-03	6.30E-03	5.50E-01	2.40E-03	7.50E-03	7.46E-01	-1.20E-02	1.15E-02	2.97E-01
rs11220462	11	126,243,952	A	G	5.90E-02	5.90E-03	6.61E-21	-1.62E-02	5.60E-03	8.75E-03	1.89E-02	5.40E-03	1.32E-03	-1.39E-02	9.30E-03	1.38E-01	-1.39E-02	1.11E-02	2.13E-01	-1.99E-02	1.70E-02	2.42E-01
rs11246602	11	51,512,090	C	T	1.90E-03	5.60E-03	5.26E-01	3.40E-02	5.20E-03	1.68E-10	-9.10E-03	5.20E-03	1.92E-01	1.37E-02	9.30E-03	1.42E-01	9.00E-03	1.11E-02	4.18E-01	1.75E-02	1.72E-02	3.08E-01
rs11563251	2	234,679,384	T	C	3.45E-02	6.20E-03	4.50E-08	5.80E-03	5.70E-03	3.65E-01	8.30E-03	5.60E-03	8.26E-02	1.36E-02	1.01E-02	1.80E-01	1.47E-02	1.20E-02	2.22E-01	1.81E-02	1.85E-02	3.28E-01
rs11660468	18	47,209,143	T	C	1.11E-02	3.70E-03	3.41E-03	3.91E-02	3.40E-03	3.60E-27	-8.00E-04	3.40E-03	8.80E-01	1.70E-03	6.40E-03	7.88E-01	1.00E-03	7.70E-03	8.96E-01	1.21E-02	1.17E-02	3.01E-01
rs1169288	12	121,416,650	C	A	3.75E-02	4.00E-03	6.45E-21	9.60E-03	3.70E-03	9.13E-03	2.50E-03	3.60E-03	4.20E-01	-1.53E-02	6.70E-03	2.25E-02	-7.50E-03	8.00E-03	3.45E-01	-3.39E-02	1.22E-02	5.47E-03
rs1186380	12	121,376,416	T	C	-2.44E-02	4.30E-03	8.46E-08	-2.00E-04	4.00E-03	8.69E-01	2.60E-03	3.90E-03	5.21E-01	1.32E-02	7.20E-03	6.74E-02	9.20E-03	8.60E-03	2.86E-01	1.46E-02	1.32E-02	2.67E-01
rs12133576	1	93,816,400	G	A	-1.04E-02	3.80E-03	3.83E-03	-2.43E-02	3.50E-03	6.15E-11	9.00E-03	3.40E-03	1.19E-02	-1.58E-02	6.60E-03	1.64E-02	-8.90E-03	7.90E-03	2.57E-01	-2.38E-02	1.20E-02	4.85E-02
rs12145743	1	156,700,651	G	T	-4.20E-03	3.90E-03	3.38E-01	2.03E-02	3.60E-03	1.80E-08	-1.23E-02	3.60E-03	5.56E-04	1.90E-03	6.60E-03	7.78E-01	-2.20E-03	7.90E-03	7.84E-01	-7.80E-03	1.22E-02	5.24E-01
rs12226802	11	55,324,308	G	A	2.00E-04	5.30E-03	6.19E-01	3.31E-02	4.90E-03	1.29E-09	-6.70E-03	5.00E-03	2.30E-01	1.17E-02	9.30E-03	2.09E-01	6.20E-03	1.11E-02	5.78E-01	1.48E-02	1.70E-02	3.84E-01
rs1250229	2	216,304,384	C	T	2.43E-02	4.20E-03	3.13E-08	-3.40E-03	3.90E-03	4.04E-01	8.90E-03	3.80E-03	1.39E-02	1.00E-04	7.10E-03	9.93E-01	5.40E-03	8.50E-03	5.23E-01	-3.20E-03	1.30E-02	8.09E-01
rs12525163	6	152,040,291	C	T	-4.30E-03	4.00E-03	2.56E-01	2.15E-02	3.70E-03	1.52E-07	-8.60E-03	3.70E-03	3.70E-02	4.78E-02	6.90E-03	3.87E-12	3.50E-02	8.20E-03	2.02E-05	7.34E-02	1.25E-02	4.03E-09
rs1260326	2	27,730,940	C	T	-2.06E-02	3.70E-03	1.51E-07	1.13E-02	3.50E-03	1.74E-03	-1.15E-01	3.40E-03	2.29E-239	1.06E-02	6.40E-03	9.48E-02	1.01E-02	7.60E-03	1.82E-01	1.02E-02	1.16E-02	3.78E-01
rs12670798	7	21,607,352	C	T	3.44E-02	4.30E-03	4.81E-14	-1.40E-03	4.00E-03	7.33E-01	1.01E-02	3.90E-03	1.68E-02	1.02E-02	7.60E-03	1.76E-01	1.79E-02	9.00E-03	4.73E-02	4.30E-03	1.38E-02	7.56E-01
rs12678919	8	19,844,222	G	A	-8.00E-03	6.10E-03	5.05E-01	1.55E-01	5.70E-03	1.38E-149	-1.70E-01	5.60E-03	1.82E-199	1.31E-02	1.04E-02	2.08E-01	1.46E-02	1.24E-02	2.37E-01	1.50E-02	1.90E-02	4.30E-01
rs12801636	11	65,391,317	A	G	7.80E-03	4.50E-03	1.45E-01	2.35E-02	4.20E-03	3.15E-08	-1.77E-02	4.00E-03	1.35E-05	-1.43E-02	7.40E-03	5.41E-02	-1.22E-02	8.90E-03	1.70E-01	-2.45E-02	1.36E-02	7.23E-02
rs13107325	4	103,188,709	T	C	-1.56E-02	8.30E-03	5.74E-02	-7.08E-02	7.80E-03	1.07E-15	3.09E-02	7.70E-03	3.98E-05	1.73E-02	1.25E-02	1.66E-01	2.01E-02	1.49E-02	1.78E-01	-6.40E-03	2.26E-02	7.76E-01
rs13326165	3	52,532,118	G	A	4.20E-03	4.60E-03	2.67E-01	-2.89E-02	4.30E-03	9.04E-11	2.05E-02	4.20E-03	2.96E-06	-2.70E-03	8.00E-03	7.31E-01	-5.70E-03	9.50E-03	5.49E-01	1.60E-03	1.46E-02	9.10E-01
rs1341267	13	95,284,980	A	C	1.60E-03	3.70E-03	8.87E-01	2.30E-03	3.40E-03	8.59E-01	-1.84E-02	3.30E-03	8.30E-07	-1.65E-02	6.50E-03	1.13E-02	-1.14E-02	7.80E-03	1.42E-01	-2.39E-02	1.18E-02	4.30E-02
rs1367117	2	21,263,900	A	G	1.19E-01	4.00E-03	9.48E-183	-2.23E-02	3.70E-03	7.59E-09	2.52E-02	3.60E-03	1.06E-11	8.00E-03	6.60E-03	2.30E-01	7.00E-04	7.90E-03	9.35E-01	2.03E-02	1.21E-02	9.33E-02
rs1482852	3	156,798,294	G	A	-2.90E-03	3.80E-03	5.75E-01	2.09E-02	3.50E-03	6.34E-08	-1.30E-02	3.50E-03	3.68E-04	-6.30E-03	6.40E-03	3.24E-01	-4.80E-03	7.60E-03	5.29E-01	-1.19E-02	1.16E-02	3.05E-01
rs1515110	2	227,122,216	T	G	6.30E-03	3.70E-03	9.36E-02	-3.23E-02	3.50E-03	8.04E-18	2.65E-02	3.40E-03	8.54E-14	4.50E-03	6.50E-03	4.87E-01	1.10E-03	7.80E-03	8.88E-01	7.50E-03	1.19E-02	5.26E-01
rs1532085	15	58,683,366	G	A	-2.60E-03	3.70E-03	6.47E-01	-1.07E-01	3.50E-03	1.24E-188	-3.10E-02	3.40E-03	2.32E-18	-5.90E-03	6.60E-03	3.72E-01	-4.50E-03	7.80E-03	5.69E-01	-5.20E-03	1.19E-02	6.65E-01
rs1535	11	61,597,972	G	A	-5.29E-02	3.80E-03	7.77E-41	-3.91E-02	3.60E-03	5.74E-27	4.63E-02	3.50E-03	5.49E-40	-7.00E-03	6.60E-03	2.90E-01	1.90E-03	7.90E-03	8.13E-01	-3.35E-02	1.21E-02	5.60E-03
rs1564348	6	160,578,860	C	T	4.81E-02	5.00E-03	2.76E-21	-7.70E-03	4.60E-03	1.68E-01	1.64E-02	4.50E-03	4.91E-04	-4.40E-03	8.40E-03	6.00E-01	-2.20E-03	1.01E-02	8.27E-01	-1.74E-02	1.54E-02	2.59E-01
rs16831243	2	135,762,344	T	C	3.78E-02	5.50E-03	9.06E-12	1.14E-02	5.10E-03	3.90E-02	-6.00E-04	5.10E-03	9.87E-01	-6.00E-04	9.70E-03	9.48E-01	6.00E-03	1.15E-02	6.04E-01	-1.39E-02	1.73E-02	4.21E-01
rs1688030	19	35,556,744	C	T	1.63E-02	7.60E-03	3.73E-02	8.50E-03	7.00E-03	2.46E-01	3.78E-02	6.80E-03	1.99E-07	1.16E-02	1.37E-02	3.96E-01	2.55E-02	1.65E-02	1.22E-01	-6.10E-03	2.48E-02	8.07E-01
rs1689797	1	182,150,978	A	C	1.42E-02	3.90E-03	4.92E-04	-3.58E-02	3.60E-03	2.85E-21	1.06E-02	3.50E-03	2.42E-02	9.00E-03	7.00E-03	1.96E-01	1.07E-02	8.30E-03	2.00E-01	9.40E-03	1.26E-02	4.55E-01
rs16942887	16	67,928,042	A	G	1.10E-03	5.40E-03	7.98E-01	8.31E-02	5.10E-03	8.28E-54	-1.15E-02	5.00E-03	2.96E-02	1.87E-02	9.40E-03	4.61E-02	1.01E-02	1.12E-02	3.65E-01	3.01E-02	1.71E-02	7.88E-02
rs17145738	7	72,982,874	T	C	3.90E-03	5.70E-03	5.43E-01	4.08E-02	5.30E-03	4.95E-13	-1.15E-01	5.30E-03	9.42E-99	3.47E-02	1.08E-02	1.29E-03	3.13E-02	1.29E-02	1.51E-02	2.84E-02	1.93E-02	1.42E-01
rs17173637	7	150,529,449	C	T	6.90E-03	6.20E-03	3.81E-01	-3.63E-02	5.70E-03	1.90E-08	2.11E-02	5.60E-03	1.04E-03	-3.73E-02	1.04E-02	3.54E-04	-3.34E-02	1.24E-02	7.04E-03	-4.86E-02	1.93E-02	1.18E-02
rs17286602	7	16,152,174	A	T	-3.20E-03	3.70E-03	4.24E-01	2.10E-02	3.40E-03	2.93E-07	-6.00E-03	3.30E-03	1.62E-01	-4.80E-03	6.60E-03	4.67E-01	-4.90E-03	7.80E-03	5.36E-01	3.00E-04	1.19E-02	9.79E-01
rs17345563	3	132,209,203	G	A	-3.58E-02	5.70E-03	2.04E-09	1.43E-02	5.30E-03	4.62E-03	-1.50E-02	5.20E-03	3.90E-03	-7.60E-03	9.80E-03	4.36E-01	-9.90E-03	1.17E-02	3.99E-01	-3.11E-02	1.79E-02	8.18E-02
rs174532	11	61,548,874	A	G	3.53E-02	4.20E-03	3.13E-16	2.12E-02	3.90E-03	6.93E-08	-1.59E-02	3.80E-03	3.44E-05	-2.30E-03	7.00E-03	7.40E-01	-4.60E-03	8.30E-03	5.76E-01	2.06E-02	1.26E-02	1.02E-01
rs17508045	2	118,576,719	C	T	-4.88E-02	6.60E-03	4.91E-12	8.50E-03	6.10E-03	4.66E-02	8.30E-03	6.00E-03	4.00E-01	2.15E-02	1.14E-02	5.95E-02	3.17E-02	1.36E-02	1.95E-02	-4.00E-03	2.08E-02	8.47E-01
rs17695224	19	52,324,216	A	G	-1.07E-02	4.20E-03	1.25E-02	-2.90E-02	3.90E-03	2.42E-13	1.18E-02	3.80E-03	1.13E-02	-9.90E-03	7.20E-03	1.70E-01	-1.39E-02	8.60E-03	1.06E-01	-2.44E-02	1.31E-02	6.30E-02
rs17788930	11	47,752,775	G	A	-4.60E-03	3.80E-03	2.18E-01	-3.59E-02	3.60E-03	1.53E-22	1.12E-02	3.50E-03	2.80E-03	4.50E-03	6.60E-03	4.90E-01	-3.00E-04	7.90E-03	9.72E-01	-6.50E-03	1.20E-02	5.89E-01
rs17789218	6	100,600,097	C	T	-2.41E-02	4.30E-03	3.26E-07	4.10E-03	4.00E-03	1.35E-01	-6.10E-03	4.00E-03	6.65E-02	9.50E-03	8.60E-03	2.67E-01	1.88E-02	1.02E-02	6.68E-02	-2.52E-02	1.57E-02	1.09E-01
rs1781930	10	5,196,273	G	A	1.02E-02	4.80E-03	5.70E-02	1.80E-03	4.50E-03	6.25E-01	3.14E-02	4.30E-03	2.51E-11	-1.51E-02	8.10E-03	6.33E-02	-2.64E-02	9.70E-03	6.54E-03	3.90E-03	1.50E-02	7.97E-01
rs1800562	6	26,093,141	A	G	-6.15E-02	8.00E-03	8.25E-14	-7.40E-03	7.40E-03	2.42E-01	1.30E-02	7.20E-03	1.72E-01	-2.90E-03	1.35E-02	8.31E-01	1.00E-02	1.62E-02	5.35E-01	-2.67E-02	2.52E-02	2.88E-01
rs1800961	20	43,042,364	T	C	-6.85E-02	1.06E-02	6.03E-10	-1.27E-01	9.90E-03	1.64E-34	-1.70E-03	9.40E-03	7.02E-01	-3.27E-02	1.75E-02	6.19E-02	-2.72E-02	2.08E-02	1.90E-01	-5.73E-02	3.34E-02	8.61E-02
rs181360	22	21,928,916	G	T	-7.40E-03	4.50E-03	7.93E-02	-3.76E-02	4.20E-03	9.24E-18	-9.10E-03	4.10E-03	2.81E-02	9.90E-03	7.60E-03	1.97E-01	1.16E-02	9.10E-03	2.02E-01	-3.10E-03	1.41E-02	8.25E-01
rs1883025	9	107,664,301	T	C	-2.96E-02	4.40E-03	6.14E-11	-6.98E-02	4.10E-03	1.50E-65	-2.19E-02	4.00E-03	2.91E-07	-1.43E-02	7.10E-03	4.40E-02	-8.90E-03	8.40E-03	2.90E-01	-4.18E-02	1.30E-02	1.27E-03
rs1998013	1	55,958,030	T	C	-3.81E-01	2.20E-02	3.02E-48	3.54E-02	2.01E-02	4.13E-01	8.90E-03	1.99E-02	6.57E-01	-4.78E-02	4.91E-02	3.30E-01	-4.00E-02	7.63E-02	6.01E-01	2.76E-02	9.07E-02	7.61E-01
rs2000999	16	72,108,093	A	G	6.50E-02	4.60E-03	4.22E-41	2.30E-03	4.30E-03	9.52E-01	1.85E-02	4.20E-03	7.49E-07	-4.20E-03	7.70E-03	5.89E-01	6.00E-03	9.10E-03	5.12E-01	-1.89E-02	1.40E-02	1.77E-01
rs2030746	2	121,309,488	T	C	2.14E-02	3.80E-03	8.61E-09	-2.50E-03	3.60E-03	3.06E-01	3.10E-03	3.50E-03	4.91E-01	1.30E-03	6.50E-03	8.36E-01	-4.00E-04	7.70E-03	9.59E-01	-5.00E-04	1.17E-02	9.66E-01
rs205262	6	34,563,164	G	A	-8.80E-03	4.10E-03	3.13E-02	-2.83E-02	3.90E-03	3.88E-13	2.80E-03	3.80E-03	8.03E-01	-9.00E-04	7.00E-03	8.99E-01	-3.00E-03	8.30E-03	7.22E-01	-5.20E-03	1.28E-02	6.87E-01
rs2068888	10	94,839,642	A	G	-1.66E-02	3.80E-03	3.89E-05	1.93E-02	3.50E-03	2.15E-06	-2.41E-02	3.40E-03	1.68E-11	-1.15E-02	6.20E-03	6.37E-02	-1.15E-02	7.40E-03	1.20E-01	2.60E-03	1.13E-02	8.15E-01
rs2073547	7	44,582,331	G	A	4.85E-02	4.90E-03	1.92E-21	-4.90E-03	4.50E-03	3.10E-01	1.48E-02	4.40E-03	3.39E-03	1.17E-02	8.40E-03	1.65E-01	1.53E-02	1.00E-02	1.26E-01	1.20E-03	1.52E-02	9.37E-01
rs217386	7	44,600,695	A	G	-3.63E-02	3.80E-03	1.20E-19	1.30E-03	3.50E-03	4.99E-01	-1.01E-02	3.40E-03	6.35E-03	-1.15E-02	6.80E-03	8.95E-02	-1.44E-02	8.10E-03	7.59E-02	-6.20E-03	1.22E-02	6.11E-01
rs2240327	3	50,113,034	G	A	5.00E-04	3.60E-03	9.71E-01	2.42E-02	3.40E-03	1.11E-11	-1.70E-03	3.30E-03	8.67E-01	-1.37E-02	6.20E-03	2.68E-02	-1.39E-02	7.40E-03	5.99E-02	-2.83E-02	1.13E-02	1.22E-02
rs2241210	12	109,950,144	G	A	7.80E-03	3.70E-03	8.55E-02	3.32E-02	3.50E-03	2.49E-20	2.90E-03	3.30E-03	2.47E-01	-1.03E-02	6.20E-03	9.59E-02	-5.20E-03	7.40E-03	4.77E-01	-1.68E-02	1.13E-02	1.36E-01
rs2247056	6	31,265,490	C	T	2.48E-02	4.30E-03	1.42E-08	1.21E-02	4.00E-03	3.79E-03	3.78E-02	3.90E-03	3.86E-21	1.00E-02	7.40E-03	1.77E-01	5.90E-03	8.90E-03	5.08E-01	2.05E-02	1.37E-02	1.35E-01
rs2255141	10	113,933,886	G	A	-2.99E-02	4.00E-03	1.32E-13	-3.37E-02	3.70E-03	2.35E-17	2.09E-02	3.60E-03	1.70E-09	1.28E-02	6.90E-03	6.45E-02	1.52E-02	8.30E-03	6.58E-02	8.60E-03	1.27E-02	4.99E-01
rs2278236	19	8,431,581	A	G	6.70E-03	3.80E-03	1.27E-01	3.31E-02	3.50E-03	3.19E-18	-1.41E-02	3.40E-03	1.52E-04	5.10E-03	6.70E-03	4.48E-01	8.00E-03	8.00E-03	3.16E-01	-1.24E-02	1.21E-02	3.05E-01
rs2287623	2	169,830,155	A	G	-2.17E-02	3.70E-03	5.40E-08	-1.12E-02	3.50E-03	2.05E-03	6.00E-04	3.40E-03	9.20E-01	2.30E-03	6.70E-03	7.28E-01	-1.10E-03	8.00E-03	8.94E-01	1.14E-02	1.21E-02	3.48E-01
rs2288002	16	72,057,282	G	A	2.85E-02	3.80E-03	1.26E-12	6.90E-03	3.50E-03	1.21E-01	8.90E-03	3.40E-03	1.69E-03	-9.00E-04	6.30E-03	8.85E-01	1.70E-03	7.40E-03	8.22E-01	-1.56E-02	1.14E-02	1.71E-01
rs2290547	3	47,061,183	A	G	6.00E-04	4.90E-03	7.93E-01	-2.97E-02	4.60E-03	3.69E-09	9.60E-03	4.40E-03	2.21E-02	-5.10E-03	8.30E-03	5.36E-01	1.30E-03	9.90E-03	8.96E-01	-1.97E-02	1.55E-02	2.04E-01
rs2293889	8	116,599,199	G	T	-1.47E-02	3.80E-03	2.42E-04	3.12E-02	3.50E-03	4.27E-17	-6.20E-03	3.40E-03	1.51E-01	-6.90E-03	6.30E-03	2.72E-01	-6.40E-03	7.50E-03	3.90E-01	3.10E-03	1.14E-02	7.84E-01
rs2294261	6	16,109,163	C	A	-3.33E-02	3.70E-03	6.57E-17	8.50E-03	3.50E-03	2.06E-02	-2.10E-03	3.40E-03	5.87E-01	-5.60E-03	6.70E-03	3.97E-01	-5.80E-03	8.00E-03	4.64E-01	9.00E-03	1.21E-02	4.55E-01
rs2297374	6	160,575,985	T	C	-3.25E-02	3.80E-03	1.26E-15	5.60E-03	3.50E-03	2.18E-01	-9.10E-03	3.40E-03	4.74E-03	-3.00E-04	6.40E-03	9.64E-01	-3.90E-03	7.60E-03	6.09E-01	3.70E-03	1.16E-02	7.48E-01
rs2303975	11	14,276,999	A	G	-1.30E-03	5.30E-03	9.35E-01	2.79E-02	4.90E-03	1.59E-07	-1.15E-02	4.80E-03	2.68E-02	2.72E-02	9.60E-03	4.55E-03	2.90E-02	1.14E-02	1.07E-02	8.20E-03	1.76E-02	6.40E-01
rs2326077	8	59,385,919	T	C	-3.41E-02	3.80E-03	5.00E-17	-4.30E-03	3.50E-03	2.18E-01	-1.80E-02	3.40E-03	5.35E-07	-1.98E-02	6.50E-03	2.42E-03	-1.83E-02	7.80E-03	1.87E-02	-4.40E-03	1.19E-02	7.14E-01
rs2328223	20	17,845,921	C	A	2.99E-02	5.00E-03	5.63E-09	-4.00E-04	4.60E-03	8.59E-01	-6.60E-03	4.50E-03	1.15E-01	-4.30E-03	8.90E-03	6.33E-01	-9.40E-03	1.07E-02	3.81E-01	-1.40E-03	1.61E-02	9.29E-01
rs2412710	15	42,683,787	A	G	-2.40E-03	1.48E-02	6.40E-01	-8.40E-02	1.39E-02	1.36E-09	9.88E-02	1.32E-02	1.66E-11	2.00E-03	2.15E-02	9.26E-01	1.48E-02	2.56E-02	5.64E-01	-7.47E-02	4.05E-02	6.52E-02
rs2472509	9	107,684,230	G	T	4.00E-04	3.90E-03	7.08E-01	2.27E-02	3.70E-03	1.21E-09	-2.40E-03	3.60E-03	7.22E-01	-1.70E-03	7.40E-03	8.15E-01	9.30E-03	8.90E-03	2.98E-01	-8.40E-03	1.34E-02	5.28E-01
rs2587534	1	234,849,339	A	G	3.91E-02	3.70E-03	8.06E-25	9.30E-03	3.40E-03	3.85E-03	4.10E-03	3.30E-03	2.71E-01	4.30E-03	6.60E-03	5.20E-01	9.20E-03	7.90E-03	2.44E-01	-4.40E-03	1.20E-02	7.13E-01
rs2602836	4	100,014,805	G	A	7.00E-04	3.70E-03	8.31E-01	-1.92E-02	3.40E-03	4.96E-08	9.00E-03	3.30E-03	2.12E-02	1.02E-02	6.40E-03	1.10E-01	1.17E-02	7.60E-03	1.25E-01	-3.70E-03	1.16E-02	7.46E-01
rs261342	15	58,731,153	C	G	2.60E-03	6.50E-03	7.36E-01	-1.07E-01	6.00E-03	1.47E-68	-4.48E-02	5.90E-03	2.53E-12	3.00E-04	7.60E-03	9.73E-01	-6.00E-04	9.00E-03	9.49E-01	3.60E-03	1.38E-02	7.95E-01
rs2642438	1	220,970,028	G	A	3.52E-02	4.20E-03	7.32E-16	3.03E-02	3.90E-03	7.78E-14	-1.72E-02	3.80E-03	5.27E-06	-8.40E-03	7.10E-03	2.32E-01	-1.24E-02	8.40E-03	1.39E-01	-1.93E-02	1.28E-02	1.34E-01
rs2652834	15	63,396,867	G	A	-1.90E-03	4.60E-03	7.32E-01	2.85E-02	4.30E-03	3.59E-11	-2.47E-02	4.20E-03	1.92E-08	3.30E-03	7.70E-03	6.66E-01	8.10E-03	9.10E-03	3.78E-01	-1.17E-02	1.39E-02	3.99E-01
rs267733	1	150,958,836	G	A	-3.31E-02	5.30E-03	5.29E-09	1.61E-02	5.00E-03	3.58E-03	-2.50E-03	4.80E-03	6.16E-01	2.16E-02	8.40E-03	1.06E-02	1.43E-02	1.01E-02	1.55E-01	3.20E-02	1.55E-02	3.88E-02
rs2710642	2	63,149,557	A	G	2.39E-02	3.80E-03	6.09E-09	-9.60E-03	3.50E-03	7.69E-03	6.50E-03	3.40E-03	4.71E-02	-1.20E-03	6.50E-03	8.58E-01	4.30E-03	7.80E-03	5.79E-01	-1.48E-02	1.18E-02	2.12E-01
rs2737252	8	116,663,898	A	G	-3.14E-02	4.10E-03	7.04E-14	-1.26E-02	3.80E-03	3.94E-03	-9.20E-03	3.70E-03	1.07E-02	-1.08E-02	6.90E-03	1.20E-01	-2.21E-02	8.30E-03	7.74E-03	-1.10E-03	1.27E-02	9.32E-01
rs2923084	11	10,388,782	G	A	1.21E-02	4.90E-03	1.84E-02	-2.56E-02	4.50E-03	5.02E-08	1.19E-02	4.40E-03	5.97E-03	-1.20E-02	8.30E-03	1.47E-01	-5.80E-03	9.90E-03	5.55E-01	-1.19E-02	1.50E-02	4.28E-01
rs2925979	16	81,534,790	C	T	3.10E-03	4.00E-03	6.30E-01	3.51E-02	3.70E-03	1.32E-19	-2.05E-02	3.60E-03	2.14E-07	8.00E-03	6.80E-03	2.40E-01	1.80E-03	8.10E-03	8.27E-01	-6.00E-04	1.24E-02	9.63E-01
rs2954022	8	126,482,621	A	C	-5.46E-02	3.60E-03	2.39E-47	3.99E-02	3.40E-03	2.12E-29	-7.80E-02	3.30E-03	2.23E-113	1.16E-02	6.20E-03	6.18E-02	1.19E-02	7.40E-03	1.10E-01	1.83E-02	1.13E-02	1.07E-01
rs2980885	8	126,474,306	A	G	-3.13E-02	4.50E-03	6.26E-11	3.54E-02	4.20E-03	1.73E-14	-5.78E-02	4.10E-03	3.00E-40	1.68E-02	8.10E-03	3.78E-02	1.12E-02	9.70E-03	2.47E-01	2.45E-02	1.47E-02	9.49E-02
rs314253	17	7,091,650	C	T	-2.42E-02	3.80E-03	3.44E-10	3.00E-03	3.50E-03	3.53E-01	-8.60E-03	3.40E-03	2.98E-02	4.10E-03	6.50E-03	5.31E-01	6.70E-03	7.70E-03	3.88E-01	5.90E-03	1.18E-02	6.19E-01
rs3198697	16	15,129,940	T	C	9.60E-03	3.70E-03	6.86E-03	1.63E-02	3.50E-03	3.28E-05	-1.98E-02	3.40E-03	2.21E-08	-9.80E-03	6.90E-03	1.55E-01	-9.10E-03	8.20E-03	2.70E-01	-7.50E-03	1.25E-02	5.47E-01
rs326214	11	47,298,360	A	G	7.10E-03	4.70E-03	2.04E-01	-6.09E-02	4.50E-03	2.17E-36	2.35E-02	4.20E-03	3.79E-07	-1.70E-02	6.70E-03	1.09E-02	-1.95E-02	7.80E-03	1.30E-02	-5.80E-03	1.22E-02	6.34E-01
rs355838	2	165,619,163	G	T	-1.78E-02	3.80E-03	3.05E-05	1.91E-02	3.50E-03	4.10E-07	-1.35E-02	3.40E-03	1.21E-04	-5.10E-03	6.50E-03	4.31E-01	-3.10E-03	7.70E-03	6.92E-01	-1.21E-02	1.17E-02	3.00E-01
rs364585	20	12,962,718	G	A	2.49E-02	3.80E-03	4.28E-10	5.00E-04	3.50E-03	8.22E-01	-1.80E-03	3.40E-03	4.40E-01	2.10E-03	6.80E-03	7.58E-01	-3.00E-03	8.20E-03	7.12E-01	5.60E-03	1.23E-02	6.48E-01
rs3741414	12	57,844,049	T	C	-1.59E-02	4.30E-03	3.41E-04	2.96E-02	4.00E-03	6.10E-14	-2.80E-02	3.90E-03	1.44E-13	1.38E-02	7.80E-03	7.84E-02	1.39E-02	9.40E-03	1.39E-01	1.30E-03	1.43E-02	9.27E-01
rs3761445	22	38,595,411	A	G	8.10E-03	3.70E-03	3.99E-02	-1.58E-02	3.50E-03	3.94E-06	2.32E-02	3.40E-03	8.06E-12	3.98E-02	6.30E-03	2.62E-10	4.49E-02	7.50E-03	2.29E-09	1.55E-02	1.15E-02	1.77E-01
rs3764351	17	37,824,339	T	C	2.10E-03	5.70E-03	7.03E-01	2.94E-02	5.40E-03	4.20E-07	-9.20E-03	5.20E-03	1.33E-01	7.60E-03	6.60E-03	2.49E-01	1.60E-02	7.90E-03	4.33E-02	-1.77E-02	1.20E-02	1.41E-01
rs3780181	9	2,640,759	G	A	-4.45E-02	7.40E-03	1.76E-09	-3.80E-03	6.80E-03	5.42E-01	6.90E-03	6.60E-03	4.91E-01	-1.34E-02	1.34E-02	3.18E-01	-2.73E-02	1.61E-02	8.86E-02	6.50E-03	2.42E-02	7.89E-01
rs3817588	2	27,731,212	C	T	-2.55E-02	4.60E-03	4.43E-07	4.90E-03	4.30E-03	2.30E-01	-6.74E-02	4.20E-03	1.30E-55	1.49E-02	8.00E-03	6.45E-02	2.22E-02	9.60E-03	2.07E-02	2.41E-02	1.47E-02	1.03E-01
rs3822072	4	89,741,269	A	G	7.40E-03	3.70E-03	3.71E-02	-2.51E-02	3.40E-03	4.06E-12	1.82E-02	3.40E-03	5.74E-07	6.00E-04	6.30E-03	9.21E-01	-3.80E-03	7.60E-03	6.12E-01	1.41E-02	1.16E-02	2.24E-01
rs38855	7	116,358,044	G	A	-1.00E-03	3.60E-03	9.73E-01	1.46E-02	3.40E-03	9.05E-05	-1.87E-02	3.30E-03	2.11E-08	5.20E-03	6.30E-03	4.04E-01	6.30E-03	7.50E-03	3.98E-01	1.80E-02	1.14E-02	1.14E-01

rs3996352	7	130,444,934	G	A	-5.30E-03	3.70E-03	1.21E-01	2.96E-02	3.40E-03	3.59E-17	-1.79E-02	3.30E-03	5.88E-08	1.77E-02	6.70E-03	8.34E-03	2.36E-02	8.00E-03	3.22E-03	1.47E-02	1.21E-02	2.24E-01
rs4075205	8	144,284,709	C	T	1.19E-02	3.80E-03	8.21E-04	-2.24E-02	3.50E-03	3.54E-09	9.00E-03	3.40E-03	5.16E-02	-1.27E-02	6.50E-03	4.92E-02	-1.48E-02	7.70E-03	5.46E-02	-1.50E-03	1.17E-02	9.01E-01
rs4148005	17	66,882,466	G	T	1.50E-02	3.90E-03	1.49E-04	-2.83E-02	3.60E-03	5.74E-14	6.60E-03	3.50E-03	4.37E-02	-1.41E-02	7.00E-03	4.42E-02	-1.52E-02	8.40E-03	7.00E-02	-2.08E-02	1.27E-02	1.03E-01
rs4148218	2	44,099,582	A	G	-4.41E-02	4.70E-03	6.76E-21	2.90E-03	4.40E-03	4.56E-01	-3.70E-03	4.20E-03	2.95E-01	1.50E-02	8.30E-03	6.94E-02	1.68E-02	9.80E-03	8.73E-02	1.77E-02	1.50E-02	2.36E-01
rs4240624	8	9,184,231	A	G	6.72E-02	6.30E-03	2.62E-23	8.18E-02	5.80E-03	1.32E-45	-2.78E-02	5.70E-03	1.09E-06	2.68E-02	1.11E-02	1.60E-02	3.40E-02	1.33E-02	1.06E-02	2.55E-02	2.04E-02	2.12E-01
rs442177	4	88,030,261	T	G	1.59E-02	3.70E-03	6.09E-05	-2.15E-02	3.40E-03	2.19E-09	3.09E-02	3.30E-03	1.32E-18	2.40E-02	6.30E-03	1.36E-04	2.60E-02	7.50E-03	5.28E-04	1.62E-02	1.14E-02	1.57E-01
rs4465830	20	44,585,420	G	A	9.00E-03	4.70E-03	5.99E-02	-5.97E-02	4.40E-03	5.18E-40	5.34E-02	4.30E-03	2.98E-34	-1.66E-02	8.00E-03	3.82E-02	-2.24E-02	9.60E-03	1.95E-02	4.20E-03	1.46E-02	7.72E-01
rs4530754	5	122,855,416	A	G	2.75E-02	3.60E-03	3.58E-12	8.00E-04	3.40E-03	9.34E-01	1.50E-03	3.30E-03	7.42E-01	5.00E-03	6.20E-03	4.16E-01	4.00E-03	7.40E-03	5.85E-01	1.09E-02	1.12E-02	3.32E-01
rs4587594	1	63,133,930	A	G	-4.93E-02	3.90E-03	1.63E-32	-1.47E-02	3.60E-03	1.08E-04	-6.94E-02	3.50E-03	3.50E-82	4.50E-03	6.60E-03	5.01E-01	5.90E-03	8.00E-03	4.60E-01	-8.90E-03	1.23E-02	4.71E-01
rs4650994	1	178,515,312	A	G	2.70E-03	3.60E-03	3.38E-01	-2.10E-02	3.40E-03	6.70E-09	2.40E-03	3.30E-03	3.98E-01	3.70E-03	6.30E-03	5.56E-01	1.30E-03	7.50E-03	8.65E-01	2.20E-03	1.14E-02	8.44E-01
rs4660293	1	40,028,180	G	A	1.05E-02	4.30E-03	1.23E-02	-3.53E-02	4.00E-03	2.86E-18	2.03E-02	3.90E-03	2.87E-07	1.47E-02	7.70E-03	5.76E-02	1.39E-02	9.30E-03	1.34E-01	2.45E-02	1.40E-02	7.98E-02
rs4722551	7	25,991,826	C	T	3.91E-02	4.90E-03	3.95E-14	1.01E-02	4.50E-03	2.47E-02	-2.67E-02	4.40E-03	1.58E-09	1.88E-02	8.90E-03	3.48E-02	2.07E-02	1.07E-02	5.18E-02	1.27E-02	1.61E-02	4.30E-01
rs4791641	17	8,161,149	T	C	-2.02E-02	3.70E-03	1.31E-07	-4.10E-03	3.40E-03	9.51E-02	2.80E-03	3.30E-03	4.59E-01	8.90E-03	6.20E-03	1.49E-01	1.47E-02	7.40E-03	4.69E-02	1.53E-02	1.13E-02	1.75E-01
rs4846914	1	230,295,691	A	G	-4.30E-03	3.70E-03	2.34E-01	4.79E-02	3.40E-03	3.51E-41	-4.01E-02	3.40E-03	7.20E-31	9.20E-03	7.00E-03	1.91E-01	1.19E-02	8.30E-03	1.54E-01	-2.57E-02	1.25E-02	4.06E-02
rs4871137	8	121,868,551	T	G	-4.30E-03	3.90E-03	2.36E-01	-2.09E-02	3.70E-03	1.93E-07	-1.30E-03	3.60E-03	6.56E-01	-7.10E-03	6.70E-03	2.89E-01	-1.62E-02	7.90E-03	4.14E-02	-7.00E-04	1.21E-02	9.52E-01
rs4917014	7	50,305,863	G	T	4.70E-03	3.90E-03	2.46E-01	2.22E-02	3.60E-03	1.03E-08	-1.20E-03	3.50E-03	8.87E-01	6.80E-03	7.40E-03	3.54E-01	1.41E-02	8.80E-03	1.10E-01	-1.16E-02	1.32E-02	3.81E-01
rs4921914	8	18,272,438	T	C	-2.29E-02	4.40E-03	1.92E-07	-1.90E-03	4.10E-03	3.94E-01	-3.53E-02	4.00E-03	4.87E-17	-9.80E-03	7.40E-03	1.85E-01	-1.17E-02	8.80E-03	1.83E-01	-1.43E-02	1.34E-02	2.88E-01
rs492571	15	44,211,273	C	T	-3.30E-03	9.70E-03	4.72E-01	-6.63E-02	9.00E-03	1.27E-12	7.99E-02	8.80E-03	6.74E-17	-1.64E-02	1.51E-02	2.79E-01	-3.40E-03	1.79E-02	8.48E-01	-7.00E-02	2.80E-02	1.23E-02
rs492602	19	49,206,417	G	A	2.93E-02	3.90E-03	9.42E-14	-3.20E-03	3.60E-03	4.27E-01	1.39E-02	3.50E-03	2.48E-04	1.16E-02	6.30E-03	6.46E-02	1.27E-02	7.50E-03	8.75E-02	-5.40E-03	1.14E-02	6.38E-01
rs4939883	18	47,167,214	C	T	2.11E-02	4.80E-03	1.47E-05	7.99E-02	4.50E-03	1.80E-66	5.20E-03	4.30E-03	3.81E-01	4.90E-03	8.20E-03	5.52E-01	9.10E-03	9.80E-03	3.57E-01	-8.70E-03	1.50E-02	5.61E-01
rs4942486	13	32,953,388	C	T	-2.43E-02	3.70E-03	2.26E-11	1.36E-02	3.40E-03	1.16E-04	-7.10E-03	3.30E-03	2.38E-02	-1.90E-02	6.20E-03	2.05E-03	-1.84E-02	7.40E-03	1.25E-02	-3.32E-02	1.12E-02	3.14E-03
rs4969178	17	76,388,202	G	A	1.10E-02	3.70E-03	8.20E-03	2.63E-02	3.50E-03	1.53E-12	-1.75E-02	3.40E-03	5.70E-06	1.14E-02	6.40E-03	7.49E-02	1.14E-02	7.70E-03	1.37E-01	1.95E-02	1.17E-02	9.56E-02
rs4976033	5	67,714,246	G	A	-1.00E-03	4.00E-03	8.75E-01	-2.15E-02	3.70E-03	6.42E-08	1.41E-02	3.60E-03	2.01E-04	1.30E-03	7.00E-03	8.48E-01	2.50E-03	8.30E-03	7.60E-01	7.30E-03	1.27E-02	5.63E-01
rs4983559	14	105,277,209	A	G	-2.60E-03	3.80E-03	5.83E-01	-1.97E-02	3.60E-03	9.57E-09	1.00E-04	3.50E-03	9.71E-01	-1.08E-02	6.40E-03	8.95E-02	-1.05E-02	7.60E-03	1.69E-01	3.60E-03	1.17E-02	7.58E-01
rs499974	11	75,455,021	A	C	1.20E-03	4.70E-03	8.26E-01	-2.63E-02	4.40E-03	1.12E-08	-9.00E-03	4.20E-03	5.41E-02	-1.20E-02	8.10E-03	1.39E-01	-2.71E-02	9.70E-03	5.05E-03	4.18E-02	1.46E-02	4.32E-03
rs515135	2	21,286,057	C	T	1.39E-01	4.80E-03	1.09E-178	-1.10E-02	4.40E-03	9.01E-03	1.92E-02	4.40E-03	1.36E-04	9.80E-03	8.30E-03	2.41E-01	-3.20E-03	9.90E-03	7.45E-01	3.34E-02	1.52E-02	2.76E-02
rs5763662	22	30,378,703	T	C	7.67E-02	1.21E-02	1.19E-08	3.33E-02	1.12E-02	6.37E-03	-1.00E-04	1.12E-02	8.88E-01	-4.64E-02	2.10E-02	2.75E-02	-7.39E-02	2.50E-02	3.14E-03	-2.23E-02	3.82E-02	5.60E-01
rs579459	9	136,154,168	C	T	6.65E-02	4.50E-03	2.42E-44	1.45E-02	4.20E-03	1.68E-03	-1.37E-02	4.10E-03	1.08E-03	3.74E-02	7.50E-03	7.44E-07	3.98E-02	9.00E-03	9.26E-06	2.76E-02	1.37E-02	4.37E-02
rs5880	16	57,015,091	C	G	4.70E-02	9.60E-03	1.59E-06	-3.07E-01	9.00E-03	1.37E-233	4.75E-02	8.50E-03	4.71E-08	-7.80E-03	1.54E-02	6.10E-01	-1.40E-02	1.84E-02	4.46E-01	6.00E-04	2.83E-02	9.83E-01
rs6016381	20	39,180,436	C	T	-3.63E-02	3.80E-03	6.85E-20	8.40E-03	3.50E-03	6.08E-02	-1.44E-02	3.40E-03	1.99E-05	9.70E-03	6.70E-03	1.45E-01	1.01E-02	8.00E-03	2.05E-01	2.43E-02	1.21E-02	4.43E-02
rs603446	11	116,654,435	T	C	-9.20E-03	3.70E-03	1.14E-02	1.80E-03	3.50E-03	8.73E-01	-5.02E-02	3.40E-03	3.92E-43	7.30E-03	6.30E-03	2.43E-01	4.10E-03	7.50E-03	5.85E-01	2.24E-02	1.14E-02	5.03E-02
rs6065311	20	39,724,338	C	T	4.17E-02	3.60E-03	1.66E-30	2.40E-03	3.40E-03	4.37E-01	6.10E-03	3.30E-03	2.27E-02	8.70E-03	6.20E-03	1.62E-01	1.25E-02	7.40E-03	8.96E-02	3.00E-04	1.13E-02	9.81E-01
rs634869	6	139,831,757	C	T	-1.27E-02	3.70E-03	8.37E-04	2.34E-02	3.40E-03	1.00E-10	-2.72E-02	3.30E-03	1.78E-14	5.00E-04	6.30E-03	9.31E-01	-5.30E-03	7.50E-03	4.81E-01	1.59E-02	1.15E-02	1.65E-01
rs6450176	5	53,298,025	A	G	1.04E-02	4.20E-03	1.18E-02	-2.54E-02	3.90E-03	6.88E-10	1.91E-02	3.80E-03	3.61E-07	1.09E-02	7.10E-03	1.24E-01	1.57E-02	8.50E-03	6.38E-02	-1.50E-03	1.30E-02	9.06E-01
rs646776	1	109,818,530	T	C	1.60E-01	4.40E-03	1.63E-272	-3.40E-02	4.10E-03	2.72E-15	3.40E-03	4.00E-03	3.73E-01	7.50E-03	7.40E-03	3.14E-01	2.00E-03	8.90E-03	8.24E-01	6.60E-03	1.36E-02	6.29E-01
rs6489818	12	112,310,580	A	G	2.81E-02	4.80E-03	4.57E-09	-4.00E-04	4.50E-03	9.28E-01	-3.60E-03	4.30E-03	5.40E-01	5.70E-03	8.10E-03	4.86E-01	-2.90E-03	9.70E-03	7.62E-01	3.30E-03	1.48E-02	8.27E-01
rs6511720	19	11,202,306	T	G	-2.21E-01	6.10E-03	3.85E-262	2.49E-02	5.70E-03	6.32E-05	-8.40E-03	5.60E-03	1.04E-01	1.40E-03	1.03E-02	8.95E-01	4.00E-04	1.23E-02	9.72E-01	2.02E-02	1.85E-02	2.77E-01
rs653178	12	112,007,756	T	C	2.27E-02	3.80E-03	3.88E-09	2.63E-02	3.50E-03	1.06E-12	-9.90E-03	3.40E-03	2.88E-02	3.11E-02	6.20E-03	5.88E-07	3.01E-02	7.40E-03	4.97E-05	3.24E-02	1.13E-02	3.98E-03
rs6544713	2	44,073,881	C	T	-8.06E-02	4.10E-03	4.84E-83	3.00E-03	3.80E-03	3.88E-01	-1.26E-02	3.70E-03	9.60E-04	1.32E-02	7.10E-03	6.46E-02	2.19E-02	8.50E-03	1.03E-02	-4.10E-03	1.28E-02	7.49E-01
rs6603981	1	92,993,807	T	C	3.40E-02	4.40E-03	3.10E-13	3.90E-03	4.20E-03	3.81E-01	7.20E-03	4.00E-03	1.74E-01	6.90E-03	7.70E-03	3.70E-01	9.90E-03	9.20E-03	2.82E-01	-1.26E-02	1.41E-02	3.71E-01
rs6680658	1	230,419,344	A	G	-5.50E-03	4.50E-03	2.18E-01	2.32E-02	4.10E-03	7.49E-08	-1.66E-02	4.00E-03	1.44E-05	2.90E-03	7.70E-03	7.07E-01	7.00E-03	9.20E-03	4.50E-01	-3.00E-03	1.40E-02	8.28E-01
rs6805251	3	119,560,606	C	T	-1.20E-02	3.70E-03	1.86E-03	-2.00E-02	3.50E-03	1.33E-08	1.10E-03	3.40E-03	9.94E-01	-7.70E-03	6.30E-03	2.24E-01	-8.60E-03	7.50E-03	2.54E-01	1.20E-03	1.15E-02	9.15E-01
rs6831256	4	3,473,139	G	A	1.88E-02	3.80E-03	9.07E-07	-1.31E-02	3.60E-03	2.97E-03	2.58E-02	3.50E-03	1.60E-12	-8.30E-03	6.50E-03	1.96E-01	-5.60E-03	7.70E-03	4.64E-01	-1.60E-02	1.17E-02	1.71E-01
rs6859	19	45,382,034	G	A	-8.35E-02	3.90E-03	4.65E-88	1.76E-02	3.60E-03	7.73E-06	-1.41E-02	3.50E-03	8.10E-05	1.04E-02	6.60E-03	1.16E-01	1.49E-02	7.90E-03	6.04E-02	2.37E-02	1.21E-02	5.07E-02
rs686030	9	15,304,782	A	C	8.50E-03	5.20E-03	2.36E-01	5.50E-02	4.90E-03	4.29E-27	2.50E-02	4.80E-03	2.23E-07	1.18E-02	9.10E-03	1.96E-01	7.00E-03	1.09E-02	5.18E-01	-3.00E-04	1.66E-02	9.84E-01
rs687339	3	135,932,359	T	C	1.10E-02	4.50E-03	9.97E-03	-3.16E-02	4.20E-03	7.11E-13	2.91E-02	4.00E-03	2.51E-12	-1.36E-02	7.40E-03	6.77E-02	-1.02E-02	8.90E-03	2.49E-01	-2.80E-02	1.35E-02	3.85E-02
rs688	19	11,227,602	T	C	5.40E-02	3.70E-03	1.01E-43	-1.06E-02	3.40E-03	1.55E-03	4.10E-03	3.40E-03	2.18E-01	6.50E-03	6.20E-03	2.97E-01	1.06E-02	7.40E-03	1.55E-01	1.02E-02	1.13E-02	3.67E-01
rs6882076	5	156,390,297	C	T	4.56E-02	3.80E-03	3.31E-31	1.50E-03	3.50E-03	6.85E-01	2.86E-02	3.50E-03	1.51E-15	-1.90E-03	6.80E-03	7.83E-01	-2.20E-03	8.10E-03	7.84E-01	-8.60E-03	1.22E-02	4.81E-01
rs702485	7	6,449,272	G	A	1.00E-03	3.70E-03	7.87E-01	2.43E-02	3.40E-03	6.45E-12	-1.60E-03	3.30E-03	4.75E-01	-3.00E-04	6.40E-03	9.62E-01	5.70E-03	7.70E-03	4.53E-01	-1.11E-02	1.17E-02	3.41E-01
rs7033354	9	16,904,846	T	C	-1.89E-02	3.80E-03	1.42E-06	1.54E-02	3.50E-03	6.54E-05	-1.90E-02	3.40E-03	4.44E-07	-1.62E-02	6.50E-03	1.26E-02	-1.82E-02	7.70E-03	1.88E-02	-7.00E-03	1.18E-02	5.53E-01
rs7117842	11	122,534,504	C	T	1.92E-02	3.80E-03	7.56E-07	2.72E-02	3.50E-03	1.06E-14	-2.00E-03	3.40E-03	5.43E-01	5.20E-03	6.80E-03	4.45E-01	9.50E-03	8.10E-03	2.43E-01	-1.14E-02	1.23E-02	3.57E-01
rs7225700	17	45,391,804	C	T	2.97E-02	3.80E-03	3.56E-13	9.80E-03	3.50E-03	2.35E-02	-4.60E-03	3.50E-03	2.36E-01	1.68E-02	6.60E-03	1.04E-02	1.51E-02	7.80E-03	5.39E-02	4.50E-03	1.19E-02	7.07E-01
rs7254892	19	45,389,596	A	G	-4.85E-01	1.19E-02	0.00E+00	5.28E-02	1.13E-02	4.17E-05	1.24E-01	1.06E-02	1.40E-24	-2.16E-02	1.70E-02	2.03E-01	-3.07E-02	2.02E-02	1.28E-01	-2.65E-02	3.16E-02	4.02E-01
rs7264396	20	34,154,741	T	C	-2.46E-02	4.50E-03	4.41E-08	-5.40E-03	4.20E-03	6.02E-02	-1.08E-02	4.10E-03	2.58E-03	3.80E-03	7.40E-03	6.07E-01	5.20E-03	8.80E-03	5.55E-01	-1.64E-02	1.34E-02	2.21E-01
rs731839	19	33,899,065	A	G	1.80E-03	4.00E-03	5.17E-01	2.20E-02	3.70E-03	3.44E-09	-2.24E-02	3.60E-03	2.65E-09	-1.71E-02	6.70E-03	1.07E-02	-2.55E-02	8.00E-03	1.41E-03	-9.30E-03	1.22E-02	4.48E-01
rs749671	16	31,088,347	A	G	-1.52E-02	3.70E-03	1.05E-04	7.10E-03	3.50E-03	9.57E-02	-2.11E-02	3.40E-03	6.11E-10	1.70E-02	6.50E-03	8.69E-03	7.20E-03	7.60E-03	3.45E-01	1.35E-02	1.17E-02	2.48E-01
rs7607980	2	165,551,201	C	T	-6.50E-03	5.60E-03	2.88E-01	4.47E-02	5.20E-03	1.81E-15	-3.62E-02	5.00E-03	2.41E-12	8.00E-03	9.20E-03	3.85E-01	1.38E-02	1.10E-02	2.09E-01	-2.22E-02	1.69E-02	1.87E-01
rs7640978	3	32,533,010	T	C	-3.92E-02	6.90E-03	9.84E-09	3.00E-04	6.40E-03	7.22E-01	-1.78E-02	6.30E-03	5.54E-03	1.82E-02	1.22E-02	1.35E-01	1.45E-02	1.45E-02	3.15E-01	1.61E-02	2.18E-02	4.60E-01
rs7703051	5	74,625,487	A	C	7.27E-02	3.70E-03	1.40E-77	2.00E-03	3.50E-03	4.21E-01	5.70E-03	3.40E-03	1.63E-01	1.35E-02	6.30E-03	3.35E-02	1.40E-02	7.60E-03	6.43E-02	1.57E-02	1.16E-02	1.75E-01
rs7832643	8	145,022,657	T	G	3.39E-02	3.80E-03	2.67E-17	-1.00E-03	3.50E-03	5.95E-01	1.70E-03	3.40E-03	4.72E-01	8.90E-03	6.50E-03	1.71E-01	7.90E-03	7.70E-03	3.10E-01	7.90E-03	1.17E-02	5.00E-01
rs7897379	10	65,301,725	C	T	1.04E-02	3.70E-03	4.07E-03	1.94E-02	3.40E-03	1.31E-08	-2.74E-02	3.30E-03	1.27E-17	-1.97E-02	6.20E-03	1.44E-03	-2.72E-02	7.40E-03	2.24E-04	1.00E-03	1.13E-02	9.30E-01
rs799160	7	73,060,006	T	C	4.50E-03	3.90E-03	2.86E-01	-1.33E-02	3.70E-03	2.94E-04	3.98E-02	3.60E-03	5.46E-30	-8.90E-03	6.90E-03	2.02E-01	-8.20E-03	8.30E-03	3.26E-01	-2.50E-03	1.25E-02	8.43E-01
rs8017377	14	24,883,887	A	G	3.03E-02	3.80E-03	2.52E-15	-3.70E-03	3.60E-03	4.34E-01	5.60E-03	3.50E-03	1.42E-01	-1.02E-02	6.70E-03	1.28E-01	-6.20E-03	8.00E-03	4.37E-01	-1.43E-02	1.21E-02	2.36E-01
rs8077889	17	41,878,166	C	A	5.00E-04	4.60E-03	9.15E-01	-2.05E-02	4.30E-03	1.50E-06	2.52E-02	4.20E-03	9.88E-09	-2.00E-03	7.70E-03	7.92E-01	-1.20E-02	9.20E-03	1.91E-01	1.24E-02	1.40E-02	3.76E-01
rs8176720	9	136,132,873	C	T	-3.32E-02	3.80E-03	1.59E-17	-5.00E-04	3.60E-03	9.43E-01	7.30E-03	3.50E-03	6.09E-02	-2.27E-02	6.60E-03	5.44E-04	-1.98E-02	7.80E-03	1.14E-02	-2.36E-02	1.19E-02	4.78E-02
rs838876	12	125,259,888	G	A	3.00E-03	4.20E-03	4.42E-01	-4.93E-02	3.90E-03	7.33E-33	5.20E-03	3.70E-03	3.77E-01	-8.70E-03	7.30E-03	2.34E-01	-1.10E-02	8.70E-03	2.07E-01	2.80E-03	1.32E-02	8.33E-01
rs868943	6	116,337,503	A	G	-2.64E-02	3.70E-03	8.44E-11	-7.50E-03	3.50E-03	3.55E-02	-1.37E-02	3.40E-03	3.18E-04	-7.00E-03	6.50E-03	2.81E-01	7.00E-04	7.70E-03	9.33E-01	-1.76E-02	1.18E-02	1.36E-01
rs894210	8	19,865,843	A	G	-7.10E-03	3.70E-03	1.22E-01	6.89E-02	3.40E-03	1.68E-84	-6.71E-02	3.30E-03	2.94E-89	8.10E-03	6.30E-03	2.02E-01	9.20E-03	7.60E-03	2.23E-01	1.02E-02	1.15E-02	3.76E-01
rs903319	1	220,985,811	C	T	2.66E-02	4.10E-03	5.22E-11	1.03E-02	3.80E-03	1.22E-02	-5.40E-03	3.70E-03	1.38E-01	-2.10E-03	7.50E-03	7.81E-01	-1.90E-03	8.90E-03	8.33E-01	-1.09E-02	1.35E-02	4.22E-01
rs9491696	6	127,452,639	G	C	5.70E-03	3.70E-03	2.64E-01	-2.03E-02	3.40E-03	5.21E-10	1.79E-02	3.40E-03	4.87E-07	-2.00E-02	6.30E-03	1.40E-03	-2.10E-02	7.50E-03	4.91E-03	-3.16E-02	1.14E-02	5.69E-03
rs952044	18	57,798,110	T	C	-3.30E-03	3.90E-03	5.79E-01	-2.31E-02	3.70E-03	1.19E-08	1.01E-02	3.60E-03	2.45E-03	-8.50E-03	6.60E-03	1.95E-01	-3.40E-03	7.90E-03	6.66E-01	-7.90E-03	1.21E-02	5.12E-01
rs9686661	5	55,861,786	T	C	1.79E-02	4.80E-03	5.29E-04	-2.83E-02	4.40E-03	1.37E-08	3.79E-02	4.40E-03	2.54E-16	-1.90E-02	8.20E-03	2.03E-02	-2.80E-02	9.80E-03	4.39E-03	-9.20E-03	1.49E-02	5.35E-01
rs9693857	8	9,267,117	T	C	-4.60E-03	3.70E-03	2.98E-01	-3.70E-03	3.50E-03	5.27E-01	2.02E-02	3.40E-03	1.69E-08	8.10E-03	7.10E-03	2.55E-01	9.70E-03	8.60E-03	2.64E-01	2.70E-02	1.30E-02	3.73E-02
rs970548	10	46,013,277	C	A	1.57E-02	4.20E-03	6.65E-04	2.58E-02	3.90E-03	1.71E-10	2.50E-03	3.80E-03	4.59E-01	1.21E-02	7.10E-03	9.03E-02	2.18E-02	8.50E-03	1.04E-02	-1.74E-02	1.31E-02	1.84E-01
rs9875338	3	12,296,469	A	G	-2.70E-02	3.70E-03	2.21E-11	-7.30E-03	3.50E-03	2.10E-02	-1.44E-02	3.40E-03	1.62E-05	-1.21E-02	6.40E-03	5.92E-02	-1.87E-02	7.60E-03	1.40E-02	6.50E-03	1.16E-02	5.74E-01
rs9930333	16	53,799,977	G	T	-2.00E-04	4.10E-03	7.18E-01	-1.99E-02	3.70E-03	2.07E-08	2.08E-02	3.70E-03	3.25E-08	-5.83E-02	6.30E-03	1.23E-20	-5.43E-02	7.40E-03	2.90E-13	-7.46E-02	1.14E-02	6.56E-11
rs998584	6	43,757,896	A	C	5.00E-04	4.00E-03	9.36E-01	-2.60E-02	3.80E-03	2.27E-11	2.93E-02	3.70E-03	3.42E-15	-2.21E-02	6.40E-03	5.46E-04	-2.53E-02	7.60E-03	8.37E-04	-1.20E-02	1.17E-02	3.06E-01
rs9989419	16	56,985,139	G	A	-2.77E-02	3.90E-03	2.49E-12	1.47E-01	3.60E-03	0.00E+00	-2.43E-02	3.50E-03	1.05E-11	7.40E-03	7.00E-03	2.94E-01	3.80E-03	8.40E-03	6.50E-01	2.45E-02	1.28E-02	5.52E-02

Supplementary Table 2

Genetic variants used in instrumental variable analysis for genes encoding drug targets.

Genetic variants used in instrumental variable analysis for genes encoding drug targets

Gene	rsid	SNP_hg19	lipid_effect_allele	lipid_other_allele	ldl_b	ldl_se	ldl_p	breast_cancer_other_allele	breast_cancer_effect_allele	bca_b	bca_se	bca_p	erpos_b	erpos_se	erpos_b	erneg_b	erneg_se	erneg_p
PCSK9	rs11206514	chr1:55516004	a	c	5.07E-02	4.10E-03	9.95E-33	C	A	7.00E-03	7.00E-03	3.16E-01	-3.90E-03	8.30E-03	6.40E-01	2.73E-02	1.25E-02	2.91E-02
PCSK9	rs11591147	chr1:55505647	g	t	4.97E-01	1.80E-02	8.58E-143	G	T	-4.42E-02	2.73E-02	1.06E-01	-2.40E-02	3.21E-02	4.55E-01	-6.22E-02	5.17E-02	2.28E-01
PCSK9	rs12067569	chr1:55528629	a	g	8.85E-02	1.00E-02	1.97E-17	G	A	2.00E-04	1.75E-02	9.90E-01	-9.70E-03	2.09E-02	6.44E-01	2.50E-03	3.18E-02	9.37E-01
PCSK9	rs2483205	chr1:55518316	c	t	5.14E-02	5.30E-03	4.74E-20	C	T	-7.80E-03	6.90E-03	2.61E-01	-8.20E-03	8.20E-03	3.14E-01	-9.40E-03	1.25E-02	4.53E-01
PCSK9	rs4927193	chr1:55509872	t	c	3.52E-02	5.60E-03	4.27E-11	T	C	-2.40E-03	9.90E-03	8.10E-01	4.20E-03	1.18E-02	7.21E-01	-1.60E-02	1.80E-02	3.74E-01
PCSK9	rs572512	chr1:55517344	t	c	4.78E-02	4.70E-03	5.31E-26	C	T	7.20E-03	7.40E-03	3.28E-01	8.20E-03	8.80E-03	3.50E-01	4.80E-03	1.34E-02	7.20E-01
PCSK9	rs7552841	chr1:55518752	t	c	3.68E-02	4.40E-03	5.40E-15	C	T	-7.10E-03	7.70E-03	3.58E-01	-6.00E-04	9.20E-03	9.51E-01	-1.11E-02	1.41E-02	4.33E-01

Gene	rsid	SNP_hg19	lipid_effect_allele	lipid_other_allele	hdl_b	hdl_se	ldl_p	breast_cancer_other_allele	breast_cancer_effect_allele	bca_b	bca_se	bca_p	erpos_b	erpos_se	erpos_b	erneg_b	erneg_se	erneg_p
CETP	rs11076174	chr16:57003146	t	c	1.80E-01	7.50E-03	2.28E-127	T	C	-2.18E-02	1.20E-02	6.79E-02	-1.86E-02	1.42E-02	1.90E-01	-3.56E-02	2.18E-02	1.03E-01
CETP	rs117040820	chr16:57005762	t	c	1.81E-01	2.16E-02	2.23E-14	C	T	4.29E-02	3.43E-02	2.12E-01	5.05E-02	4.11E-02	2.20E-01	-3.30E-03	6.41E-02	9.59E-01
CETP	rs118146573	chr16:57000938	g	a	2.62E-01	7.40E-03	2.03E-236	G	A	-4.30E-03	1.01E-02	6.68E-01	-1.40E-03	1.20E-02	9.06E-01	-2.63E-02	1.85E-02	1.54E-01
CETP	rs12597002	chr16:57002404	c	a	8.49E-02	3.90E-03	1.13E-102	C	A	-1.33E-02	6.80E-03	4.98E-02	-2.07E-02	8.00E-03	1.03E-02	5.00E-03	1.23E-02	6.85E-01
CETP	rs12720873	chr16:57006072	a	g	9.54E-02	1.39E-02	1.88E-10	G	A	1.51E-02	1.91E-02	4.29E-01	1.28E-02	2.27E-02	5.73E-01	2.54E-02	3.54E-02	4.73E-01
CETP	rs1864163	chr16:56997233	g	a	2.25E-01	4.20E-03	0.00E+00	G	A	-6.90E-03	7.20E-03	3.35E-01	-7.70E-03	8.60E-03	3.71E-01	-1.41E-02	1.31E-02	2.80E-01
CETP	rs289714	chr16:57007451	a	g	2.14E-01	5.00E-03	0.00E+00	G	A	5.80E-03	8.60E-03	4.99E-01	2.20E-03	1.02E-02	8.33E-01	1.30E-02	1.56E-02	4.05E-01
CETP	rs4784745	chr16:57014875	a	g	8.04E-02	5.50E-03	3.50E-46	A	G	-1.47E-02	6.50E-03	2.46E-02	-1.46E-02	7.80E-03	6.11E-02	-1.25E-02	1.19E-02	2.94E-01
CETP	rs5880	chr16:57015091	g	c	3.07E-01	9.00E-03	1.37E-233	G	C	-7.80E-03	1.54E-02	6.10E-01	-1.40E-02	1.84E-02	4.46E-01	6.00E-04	2.83E-02	9.83E-01
CETP	rs5883	chr16:57007353	t	c	1.15E-01	8.40E-03	1.76E-31	C	T	1.44E-02	1.43E-02	3.13E-01	2.35E-02	1.70E-02	1.65E-01	-1.41E-02	2.63E-02	5.93E-01
CETP	rs9923854	chr16:57017002	g	t	8.44E-02	9.70E-03	1.53E-15	T	G	9.00E-04	1.06E-02	9.34E-01	7.20E-03	1.26E-02	5.71E-01	-3.00E-04	1.93E-02	9.88E-01

Gene	rsid	SNP_hg19	lipid_effect_allele	lipid_other_allele	ldl_b	ldl_se	ldl_p	breast_cancer_other_allele	breast_cancer_effect_allele	bca_b	bca_se	bca_p	erpos_b	erpos_se	erpos_b	erneg_b	erneg_se	erneg_p
NPC1L1	rs17655652	chr7:44580991	t	c	2.80E-02	4.40E-03	2.18E-10	T	C	-1.43E-02	7.70E-03	6.29E-02	-1.91E-02	9.30E-03	3.93E-02	-7.90E-03	1.40E-02	5.70E-01
NPC1L1	rs2072183	chr7:44579180	c	g	3.86E-02	4.70E-03	7.12E-16	G	C	7.60E-03	8.00E-03	3.44E-01	1.06E-02	9.50E-03	2.67E-01	-2.30E-03	1.45E-02	8.74E-01
NPC1L1	rs217406	chr7:44573761	g	c	3.88E-02	5.20E-03	4.09E-14	C	G	1.30E-02	9.00E-03	1.49E-01	1.75E-02	1.08E-02	1.04E-01	-5.50E-03	1.65E-02	7.40E-01

Gene	rsid	SNP_hg19	lipid_effect_allele	lipid_other_allele	ldl_b	ldl_se	ldl_p	breast_cancer_other_allele	breast_cancer_effect_allele	bca_b	bca_se	bca_p	erpos_b	erpos_se	erpos_b	erneg_b	erneg_se	erneg_p
LDLR	rs2738464	chr19:11242307	c	g	4.22E-02	6.10E-03	2.73E-10	G	C	-8.80E-03	9.90E-03	3.74E-01	-1.14E-02	1.18E-02	3.37E-01	3.10E-03	1.81E-02	8.65E-01
LDLR	rs5742911	chr19:11243445	a	g	6.06E-02	5.70E-03	4.83E-24	A	G	-8.00E-03	6.90E-03	2.46E-01	-1.04E-02	8.20E-03	2.03E-01	4.00E-03	1.25E-02	7.50E-01
LDLR	rs6511720	chr19:11202306	g	t	2.21E-01	6.10E-03	3.85E-262	G	T	1.40E-03	1.03E-02	8.95E-01	4.00E-04	1.23E-02	9.72E-01	2.02E-02	1.85E-02	2.77E-01
LDLR	rs6511721	chr19:11206575	g	a	6.06E-02	5.10E-03	1.35E-29	G	A	-5.70E-03	6.90E-03	4.08E-01	-7.90E-03	8.20E-03	3.31E-01	3.20E-03	1.24E-02	8.00E-01
LDLR	rs688	chr19:11227602	t	c	5.40E-02	3.70E-03	1.01E-43	C	T	6.50E-03	6.20E-03	2.97E-01	1.06E-02	7.40E-03	1.55E-01	1.02E-02	1.13E-02	3.67E-01
LDLR	rs73015030	chr19:11207516	g	a	1.52E-01	1.48E-02	2.62E-22	G	A	2.29E-02	2.04E-02	2.62E-01	3.05E-02	2.45E-02	2.13E-01	9.60E-03	3.73E-02	7.96E-01

Gene	rsid	SNP_hg19	lipid_effect_allele	lipid_other_allele	ldl_b	ldl_se	ldl_p	breast_cancer_other_allele	breast_cancer_effect_allele	bca_b	bca_se	bca_p	erpos_b	erpos_se	erpos_b	erneg_b	erneg_se	erneg_p
HMGCR	rs10515198	chr5:74641560	a	g	5.99E-02	6.10E-03	5.99E-22	G	A	2.50E-03	1.03E-02	8.10E-01	-7.40E-03	1.23E-02	5.48E-01	-5.00E-03	1.88E-02	7.92E-01
HMGCR	rs12916	chr5:74656539	c	t	7.33E-02	3.80E-03	7.79E-78	T	C	1.13E-02	6.30E-03	7.17E-02	1.36E-02	7.50E-03	6.89E-02	1.05E-02	1.15E-02	3.60E-01
HMGCR	rs2303152	chr5:74641707	a	g	4.23E-02	6.40E-03	1.04E-09	G	A	7.70E-03	1.02E-02	4.48E-01	2.22E-02	1.21E-02	6.72E-02	5.00E-04	1.86E-02	9.79E-01

Supplementary Table 3

Mendelian randomization results using genetic variants associated with blood lipid levels ($P < 5x10^{-8}$) as instrumental variables.

Mendelian randomization results using all independent genetic variants associated with blood lipid levels (*P*<5e-08) as instrumental variables for low-density lipoprotein-cholesterol (LDL), high-density lipoprotein-cholesterol (HDL) and triglycerides (TG). Outcomes are risk of any breast cancer (BCA), risk of estrogen receptor positive (ERpos) and negative (ERneg) breast cancer.

	LDL	b	se	95% lci	95% uci	p	OR	95% LCI	95% UCI	Q' p-value
BCA	IVW	0.048	0.025	-0.001	0.098	0.055	1.050	0.999	1.103	3.33E-16
	Egger	0.004	0.039	-0.073	0.081	0.917	1.004	0.930	1.084	
	Intercept	0.003	0.002	-0.001	0.008	0.140	1.003	0.999	1.008	
	Median	0.045	0.025	-0.003	0.094	0.064	1.046	0.997	1.098	
Erpos	IVW	0.041	0.028	-0.014	0.095	0.144	1.041	0.986	1.100	1.59E-11
	Egger	-0.022	0.043	-0.106	0.062	0.604	0.978	0.899	1.064	
	Intercept	0.005	0.002	0.000	0.010	0.056	1.005	1.000	1.010	
	Median	0.010	0.029	-0.047	0.066	0.742	1.010	0.954	1.068	
Erneg	IVW	0.037	0.037	-0.036	0.109	0.321	1.037	0.965	1.115	5.19E-06
	Egger	0.028	0.058	-0.086	0.143	0.629	1.029	0.917	1.153	
	Intercept	0.001	0.003	-0.006	0.007	0.851	1.001	0.994	1.007	
	Median	0.051	0.047	-0.040	0.143	0.271	1.053	0.961	1.154	

	HDL	b	se	95% lci	95% uci	p	OR	95% LCI	95% UCI	Q' p-value
BCA	IVW	0.096	0.032	0.034	0.158	0.003	1.101	1.034	1.171	7.06E-26
	Egger	0.056	0.053	-0.049	0.160	0.295	1.057	0.953	1.174	
	Intercept	0.002	0.003	-0.003	0.007	0.350	1.002	0.997	1.007	
	Median	0.054	0.027	0.001	0.108	0.047	1.056	1.001	1.114	
Erpos	IVW	0.103	0.034	0.037	0.169	0.002	1.109	1.038	1.185	9.68E-17
	Egger	0.048	0.057	-0.063	0.159	0.401	1.049	0.939	1.172	
	Intercept	0.003	0.003	-0.002	0.008	0.221	1.003	0.998	1.008	
	Median	0.048	0.032	-0.013	0.110	0.125	1.050	0.987	1.116	
Erneg	IVW	0.086	0.046	-0.004	0.177	0.062	1.090	0.996	1.194	2.40E-10
	Egger	0.108	0.078	-0.045	0.262	0.166	1.114	0.956	1.299	
	Intercept	-0.001	0.004	-0.008	0.006	0.725	0.999	0.992	1.006	
	Median	0.051	0.049	-0.046	0.148	0.303	1.052	0.955	1.159	

	TG	b	se	95% lci	95% uci	p	OR	95% LCI	95% UCI	Q' p-value
BCA	IVW	-0.096	0.043	-0.181	-0.011	0.026	0.908	0.834	0.989	6.19E-26
	Egger	-0.124	0.073	-0.266	0.019	0.089	0.884	0.766	1.019	
	Intercept	0.002	0.004	-0.006	0.009	0.636	1.002	0.994	1.009	
	Median	-0.093	0.031	-0.154	-0.033	0.002	0.911	0.857	0.967	
Erpos	IVW	-0.099	0.047	-0.191	-0.007	0.036	0.906	0.826	0.993	3.99E-19
	Egger	-0.139	0.079	-0.293	0.016	0.078	0.871	0.746	1.016	
	Intercept	0.003	0.004	-0.005	0.011	0.529	1.003	0.995	1.011	
	Median	-0.087	0.036	-0.158	-0.017	0.015	0.916	0.854	0.983	
Erneg	IVW	-0.106	0.055	-0.213	0.001	0.052	0.899	0.808	1.001	6.99E-07
	Egger	-0.094	0.092	-0.274	0.086	0.308	0.911	0.761	1.090	
	Intercept	-0.001	0.005	-0.010	0.009	0.865	0.999	0.990	1.009	
	Median	-0.089	0.057	-0.201	0.023	0.121	0.915	0.818	1.024	

IVW Inverse variance-weighted method

Egger MR Egger method

Intercept Intercept term from MR Egger regression

Median Weighted Median method. Bowden et al. Genet Epidemiol 2016;40(4):304-314. PMID 27061298.

Q' p-value Q' heterogeneity test using modified second order weighting for two-sample Mendelian randomization studies. Bowden et al. Improving the accuracy of two-sample summary data Mendelian randomization: moving beyond the NOME assumption. Preprint manuscript posted on biorxiv February 27, 2018. doi: https://doi.org/10.1101/159442

Supplementary Table 4

Mendelian randomization results using genetic variants associated with blood lipid levels (*P* < $5x10^{-8}$) as instrumental variables. For each lipid, all variants associated with any of the other two lipids (*P* < 0.001) have been excluded.

Mendelian randomization results using independent genetic variants associated with blood lipid levels (*P*<5e-08) as instrumental variables for low-density lipoprotein-cholesterol (LDL), high-density lipoprotein-cholesterol (HDL) and triglycerides (TG). Outcomes are risk of any breast cancer (BCA), risk of estrogen receptor positive (ERpos) and negative (ERneg) breast cancer. For each lipid, all variants that are also associated with any of the other two lipids (*P*<0.001) have been excluded.

	LDL	b	se	95% lci	95% uci	p	OR	95% LCI	95% UCI	Q' p-value
BCA	IVW	0.114	0.048	0.020	0.208	0.017	1.121	1.021	1.231	3.97E-08
	Egger	0.169	0.129	-0.084	0.422	0.191	1.184	0.919	1.525	
	Intercept	-0.002	0.005	-0.012	0.007	0.647	0.998	0.988	1.007	
	Median	0.148	0.048	0.054	0.243	0.002	1.160	1.055	1.275	
Erpos	IVW	0.153	0.053	0.050	0.257	0.004	1.166	1.051	1.293	1.08E-05
	Egger	0.165	0.152	-0.133	0.463	0.278	1.179	0.876	1.588	
	Intercept	0.000	0.006	-0.012	0.011	0.934	1.000	0.988	1.011	
	Median	0.193	0.059	0.077	0.309	0.001	1.213	1.080	1.362	
Erneg	IVW	0.028	0.064	-0.097	0.152	0.663	1.028	0.908	1.165	0.041
	Egger	0.130	0.173	-0.209	0.469	0.451	1.139	0.812	1.598	
	Intercept	-0.004	0.007	-0.017	0.009	0.523	0.996	0.983	1.009	
	Median	0.034	0.083	-0.129	0.196	0.685	1.034	0.879	1.216	

	HDL	b	se	95% lci	95% uci	p	OR	95% LCI	95% UCI	Q' p-value
BCA	IVW	0.075	0.058	-0.039	0.189	0.198	1.078	0.962	1.208	0.003
	Egger	0.117	0.168	-0.212	0.447	0.485	1.125	0.809	1.563	
	Intercept	-0.001	0.005	-0.012	0.009	0.787	0.999	0.988	1.009	
	Median	0.085	0.066	-0.045	0.214	0.200	1.088	0.956	1.239	
Erpos	IVW	0.123	0.056	0.013	0.233	0.028	1.131	1.014	1.263	0.169
	Egger	0.053	0.161	-0.263	0.369	0.742	1.054	0.769	1.446	
	Intercept	0.002	0.005	-0.008	0.012	0.641	1.002	0.992	1.012	
	Median	0.119	0.073	-0.024	0.262	0.102	1.127	0.977	1.300	
Erneg	IVW	0.015	0.106	-0.192	0.221	0.890	1.015	0.825	1.248	0.003
	Egger	0.466	0.289	-0.101	1.032	0.108	1.593	0.904	2.808	
	Intercept	-0.015	0.009	-0.033	0.003	0.096	0.985	0.967	1.003	
	Median	0.046	0.118	-0.185	0.277	0.697	1.047	0.831	1.319	

	TG	b	se	95% lci	95% uci	p	OR	95% LCI	95% UCI	Q' p-value
BCA	IVW	-0.110	0.133	-0.371	0.151	0.407	0.896	0.690	1.163	0.153
	Egger	-0.463	0.401	-1.248	0.323	0.248	0.630	0.287	1.381	
	Intercept	0.013	0.014	-0.015	0.041	0.350	1.013	0.985	1.042	
	Median	-0.120	0.117	-0.349	0.108	0.302	0.887	0.705	1.114	
Erpos	IVW	-0.111	0.202	-0.507	0.285	0.583	0.895	0.602	1.330	0.036
	Egger	-0.361	0.707	-1.747	1.025	0.610	0.697	0.174	2.788	
	Intercept	0.009	0.025	-0.040	0.059	0.707	1.010	0.961	1.061	
	Median	-0.058	0.140	-0.332	0.216	0.679	0.944	0.717	1.242	
Erneg	IVW	-0.151	0.290	-0.719	0.418	0.603	0.860	0.487	1.519	0.056
	Egger	-1.478	0.537	-2.529	-0.426	0.006	0.228	0.080	0.653	
	Intercept	0.050	0.019	0.013	0.088	0.009	1.052	1.013	1.092	
	Median	-0.200	0.207	-0.607	0.206	0.335	0.819	0.545	1.229	

IVW Inverse variance-weighted method

Egger MR Egger method

Intercept Intercept term from MR Egger regression

Median Weighted Median method. Bowden et al. Genet Epidemiol 2016;40(4):304-314. PMID 27061298.

Q' p-value Q' heterogeneity test using modified second order weighting for two-sample Mendelian randomization studies. Bowden et al. Improving the accuracy of two-sample summary data Mendelian randomization: moving beyond the NOME assumption. Preprint manuscript posted on biorxiv February 27, 2018. doi: https://doi.org/10.1101/159442

Supplementary Table 5

Results from MR-PRESSO applied after excluding genetic variants associated with other lipids (*P* < 0.001).

MR-PRESSO (Mendelian randomization pleiotropy residual sum and outlier) results using independent genetic variants associated with blood lipid levels (P<5e-08) as instrumental variables for low-density lipoprotein-cholesterol (LDL), high-density lipoprotein-cholesterol (HDL) and triglycerides (TG). Outcomes are risk of any breast cancer (BCA), risk of estrogen receptor positive (ERpos) and negative (ERneg) breast cancer. For each lipid, all variants that are also associated with any of the other two lipids (P<0.001) have been excluded.

		Causal estimate	SE	P-value	OR	95% LCI	95% UCI	MR-PRESSO global heterogeneity p-value	Q' p-value
	**LDL**								
**BCA**	**IVW (initial)**	0.114	0.048	0.021	1.121	1.021	1.231	<0.001	3.97E-08
	**IVW (after outlier removal)**	0.090	0.037	0.020	1.094	1.018	1.176	0.109	0.102
**ERPos**	**IVW (initial)**	0.153	0.053	0.006	1.166	1.051	1.293	<0.001	1.08E-05
	**IVW (after outlier removal)**	0.129	0.043	0.004	1.138	1.047	1.237	0.125	0.124
**Erneg**	**IVW (initial)**	0.028	0.064	0.666	1.028	0.908	1.165	0.048	0.041
	**IVW (after outlier removal)**	0.031	0.055	0.577	1.032	0.926	1.150	0.438	0.438
	**HDL**								
**BCA**	**IVW (initial)**	0.075	0.058	0.209	1.078	0.962	1.208	0.002	0.003
	**IVW (after outlier removal)**	0.072	0.051	0.171	1.074	0.973	1.187	0.092	0.090
**Erpos**	**IVW (initial)**	0.123	0.056	0.037	1.131	1.014	1.263	0.202	0.169
		-	-	-	-	-	-	-	-
**Erneg**	**IVW (initial)**	0.015	0.106	0.891	1.015	0.825	1.248	0.003	0.003
	**IVW (after outlier removal)**	0.084	0.091	0.365	1.088	0.910	1.301	0.115	0.108
	**TG***								
**BCA**	**IVW (initial)**	-0.110	0.133	0.468	0.896	0.690	1.163	0.316	0.153
		-	-	-	-	-	-	-	-
**Erpos**	**IVW (initial)**	-0.111	0.202	0.621	0.895	0.602	1.330	0.149	0.036
		-	-	-	-	-	-	-	-
**Erneg**	**IVW (initial)**	-0.151	0.290	0.639	0.860	0.487	1.519	0.037	0.056
		-	-	-	-	-	-	-	-

*(too few variants to exclude outliers)

**IVW (initial)**	Inverse variance-weighted analysis
**IVW (after outlier removal)**	Inverse variance-weighted analysis after removal of outlying variants identified by the MR-PRESSO heterogeneity test
**Q' p-value**	Q' heterogeneity test using modified second order weighting for two-sample Mendelian randomization studies. Bowden et al. Improving the accuracy of two-sample summary data Mendelian randomization: moving beyond the NOME assumption. Preprint manuscript posted on biorxiv February 27, 2018. doi: https://doi.org/10.1101/159442
**MR-PRESSO global heterogeneity p-value**	MR-PRESSO global heterogeneity test. Verbanck et al. Nat Genet 2018;50(5):693-698. PMID: 29686387.

Supplementary Table 6

Results from MR-PRESSO applied without excluding genetic variants associated with other lipids.

MR-PRESSO (Mendelian randomization pleiotropy residual sum and outlier) results using independent genetic variants associated with blood lipid levels (P<5e-08) as instrumental variables for low-density lipoprotein-cholesterol (LDL), high-density lipoprotein-cholesterol (HDL) and triglycerides (TG). Outcomes are risk of any breast cancer (BCA), risk of estrogen receptor positive (ERpos) and negative (ERneg) breast cancer.

		Causal estimate	SE	P-value	OR	95% LCI	95% UCI	MR-PRESSO global heterogeneity p-value	Q' p-value
	**LDL**								
**BCA**	**IVW (initial)**	0.048	0.025	0.058	1.050	0.999	1.103	<2e-04	3.32E-16
	**IVW (after outlier removal)**	0.028	0.022	0.198	1.028	0.986	1.073	<2e-04	2.79E-07
**ERPos**	**IVW (initial)**	0.041	0.028	0.148	1.041	0.986	1.100	<2e-04	1.59E-11
	**IVW (after outlier removal)**	0.022	0.025	0.364	1.023	0.975	1.073	<2e-04	7.34E-06
**Erneg**	**IVW (initial)**	0.037	0.037	0.325	1.037	0.965	1.115	<2e-04	5.19E-06
	**IVW (after outlier removal)**	0.031	0.036	0.389	1.032	0.961	1.107	<2e-04	4.58E-05
	**HDL**								
**BCA**	**IVW (initial)**	0.096	0.032	0.003	1.101	1.034	1.171	<2e-04	7.00E-26
	**IVW (after outlier removal)**	0.086	0.025	0.001	1.090	1.037	1.145	<2e-04	1.44E-09
**Erpos**	**IVW (initial)**	0.103	0.034	0.003	1.109	1.038	1.185	<2e-04	9.64E-17
		0.099	0.027	0.001	1.104	1.046	1.164	<2e-04	1.05E-05
**Erneg**	**IVW (initial)**	0.086	0.046	0.066	1.090	0.996	1.194	<2e-04	2.40E-10
	**IVW (after outlier removal)**	0.076	0.041	0.071	1.079	0.995	1.170	<2e-04	8.65E-06
	**TG**								
**BCA**	**IVW (initial)**	-0.096	0.043	0.031	0.908	0.834	0.989	<2e-04	6.14E-26
		-0.109	0.027	1.72E-04	0.897	0.851	0.945	0.001	1.31E-03
**Erpos**	**IVW (initial)**	-0.099	0.047	0.041	0.906	0.826	0.993	<2e-04	3.98E-19
		-0.122	0.028	8.96E-05	0.885	0.837	0.936	0.051	4.52E-02
**Erneg**	**IVW (initial)**	-0.106	0.055	0.058	0.899	0.808	1.001	<2e-04	6.99E-07
		-0.091	0.044	0.046	0.913	0.837	0.996	0.051	1.36E-02

IVW (initial) — Inverse variance-weighted analysis

IVW (after outlier removal) — Inverse variance-weighted analysis after removal of outlying variants identified by the MR-PRESSO heterogeneity test

Q' p-value — Q' heterogeneity test using modified second order weighting for two-sample Mendelian randomization studies. Bowden et al. Improving the accuracy of two-sample summary data Mendelian randomization: moving beyond the NOME assumption. Preprint manuscript posted on biorxiv February 27, 2018. doi: https://doi.org/10.1101/159442

MR-PRESSO global heterogeneity p-value — MR-PRESSO global heterogeneity test. Verbanck et al. Nat Genet 2018;50(5):693-698. PMID: 29686387.

Supplementary Table 7

Mendelian randomization results of changes in blood lipids due to genetic variants in genes encoding drug targets.

The analysis takes into account the correlation between variants (R2<0.4). References: Yavorska & Burgess Int J Epidemiol 2017;46(6):1734-1739. PMID: 28398548. Burgess et al. Stat Med 2016;35(11):1880-1906. PMID: 2661904.

Genetic correlations considered in MR		b	se	95% lci	95% uci	p	OR	95% LCI	95% UCI	Q' p-value
	**PCSK9**									
	**IVW**	0.095	0.039	0.019	0.172	0.014	1.100	1.019	1.187	0.867
**BCA**	**Egger**	0.090	0.083	-0.073	0.253	0.281	1.094	0.929	1.288	
	**Egger intercept**	0.000	0.005	-0.010	0.011	0.928	1.000	0.990	1.011	
	**IVW**	0.076	0.046	-0.014	0.166	0.099	1.079	0.986	1.181	0.893
**Erpos**	**Egger**	0.055	0.086	-0.113	0.223	0.523	1.056	0.893	1.249	
	**Egger intercept**	0.002	0.006	-0.009	0.013	0.739	1.002	0.991	1.013	
	**IVW**	0.123	0.072	-0.019	0.264	0.089	1.130	0.982	1.302	0.619
**Erneg**	**Egger**	0.102	0.114	-0.120	0.325	0.368	1.108	0.887	1.384	
	**Egger intercept**	0.002	0.007	-0.012	0.016	0.815	1.002	0.988	1.016	
	**CETP**									
	**IVW**	0.065	0.020	0.026	0.105	0.001	1.068	1.026	1.110	0.530
**BCA**	**Egger**	-0.003	0.072	-0.143	0.138	0.972	0.997	0.866	1.148	
	**Egger intercept**	0.011	0.010	-0.010	0.031	0.311	1.011	0.990	1.032	
	**IVW**	0.078	0.024	0.031	0.124	0.001	1.081	1.032	1.133	0.390
**Erpos**	**Egger**	0.005	0.077	-0.147	0.157	0.948	1.005	0.864	1.170	
	**Egger intercept**	0.011	0.011	-0.011	0.033	0.315	1.011	0.989	1.034	
	**IVW**	0.065	0.037	-0.007	0.137	0.075	1.067	0.993	1.147	0.938
**Erneg**	**Egger**	0.034	0.103	-0.167	0.235	0.741	1.035	0.846	1.266	
	**Egger intercept**	0.005	0.015	-0.024	0.034	0.746	1.005	0.976	1.035	
	**NPC1L1**									
	**IVW**	0.334	0.131	0.076	0.591	0.011	1.396	1.079	1.806	0.667
**BCA**	**Egger**	-0.329	1.655	-3.573	2.914	0.842	0.719	0.028	18.433	
	**Egger intercept**	0.022	0.056	-0.087	0.131	0.687	1.023	0.917	1.140	
	**IVW**	0.449	0.157	0.141	0.757	0.004	1.567	1.151	2.133	0.625
**Erpos**	**Egger**	-0.423	1.860	-4.068	3.222	0.820	0.655	0.017	25.089	
	**Egger intercept**	0.030	0.063	-0.093	0.152	0.637	1.030	0.911	1.164	
	**IVW**	0.057	0.239	-0.411	0.525	0.811	1.059	0.663	1.691	0.797
**Erneg**	**Egger**	-1.088	1.660	-4.341	2.164	0.512	0.337	0.013	8.709	
	**Egger intercept**	0.038	0.058	-0.076	0.152	0.509	1.039	0.927	1.165	
	**LDLR**									
	**IVW**	0.012	0.036	-0.057	0.082	0.726	1.013	0.944	1.086	0.413
**BCA**	**Egger**	-0.034	0.053	-0.139	0.070	0.522	0.966	0.871	1.073	
	**Egger intercept**	0.005	0.004	-0.003	0.013	0.231	1.005	0.997	1.013	
	**IVW**	0.023	0.049	-0.074	0.119	0.642	1.023	0.929	1.127	0.249
**Erpos**	**Egger**	-0.045	0.063	-0.169	0.079	0.475	0.956	0.844	1.082	
	**Egger intercept**	0.007	0.005	-0.002	0.017	0.141	1.007	0.998	1.017	
	**IVW**	-0.070	0.063	-0.193	0.052	0.260	0.932	0.824	1.053	0.897
**Erneg**	**Egger**	-0.178	0.145	-0.462	0.106	0.220	0.837	0.630	1.112	
	**Egger intercept**	0.012	0.012	-0.011	0.035	0.309	1.012	0.989	1.035	
	**HMGCR**									
	**IVW**	0.146	0.085	-0.021	0.313	0.086	1.157	0.980	1.367	0.827
**BCA**	**Egger**	0.224	0.398	-0.557	1.005	0.574	1.251	0.573	2.731	
	**Egger intercept**	-0.006	0.027	-0.059	0.048	0.835	0.994	0.942	1.049	
	**IVW**	0.159	0.101	-0.039	0.357	0.116	1.172	0.961	1.429	0.168
**Erpos**	**Egger**	0.088	0.391	-0.677	0.854	0.822	1.092	0.508	2.348	
	**Egger intercept**	0.005	0.027	-0.047	0.058	0.847	1.005	0.954	1.059	
	**IVW**	0.130	0.155	-0.174	0.434	0.402	1.139	0.840	1.543	0.798
**Erneg**	**Egger**	0.489	0.859	-1.196	2.173	0.570	1.630	0.302	8.788	
	**Egger intercept**	-0.026	0.059	-0.142	0.089	0.656	0.974	0.868	1.093	
